# Atypical case of posterior reversible encephalopathy syndrome related to late onset postpartum eclampsia: A case report

**DOI:** 10.1097/MD.0000000000015187

**Published:** 2019-04-19

**Authors:** Kaori Masai, Yayoi Ueda, Hiromichi Naito, Kohei Tsukahara, Toshiyuki Aokage, Noritomo Fujisaki, Hirotsugu Yamamoto, Atsunori Nakao

**Affiliations:** Advanced Emergency and Critical Care Medical Center, Okayama University Hospital, Okayama City, Okayama, Japan.

**Keywords:** posterior reversible encephalopathy syndrome, postpartum eclampsia

## Abstract

**Rationale:**

Eclampsia, an obstetric emergency frequently seen in pregnant or puerperal women, is a risk factor for posterior reversible encephalopathy syndrome (PRES). Most cases of eclampsia occur postpartum. We report a woman with PRES associated with eclampsia 10 weeks post-delivery, the latest onset ever reported.

**Patient concerns:**

A 23-year-old healthy woman presented headache and nausea 10 weeks after delivery. Two days later, she generalized tonic-clonic seizure. Her brain MRI presented the foci which is typical of PRES.

**Diagnosis:**

The patient was diagnosed as PRES associated with eclampsia.

**Interventions:**

The patient received levetiracetam and edaravone.

**Outcomes:**

Her clinical course was uneventful and she fully recovered without neurological complications

**Lessons:**

The possible diagnosis of late onset postpartum eclampsia, even weeks post-delivery, should be considered, since initiation of early treatment averts severe complications and decreases mortality. Sharing our experience may increase awareness of PRES induced by late-onset postpartum eclampsia.

## 1 Introduction

Posterior reversible encephalopathy syndrome (PRES) is a clinical syndrome with distinctive imaging features marked by visual impairment, acute headaches, seizures, vomiting, altered mental status, and focal neurologic deficit.^[1,2]^ Radiologically, PRES typically shows vasogenic edema predominantly involving the bilateral parieto-occipital regions in the brain. Although PRES is known to have “reversible” symptoms and foci, it occasionally progresses to cytotoxic edema, which is irreversible and has sequelae. Prompt diagnosis of PRES using magnetic resonance imaging (MRI), immediate elimination of the causes, and antihypertensive and anticonvulsant therapy prevent serious complications.^[3,4]^ The pathogenesis of PRES is still not fully understood, although it seems to be linked to failure of local blood flow autoregulation and endothelial dysfunction.^[5]^ Several circumstances can spark the condition; the most common factors include abnormal kidney function, acute blood pressure elevation, transplantation, systemic infections, chemotherapy, neoplasia treatment, and chronic or acute kidney disease.^[6]^ In addition, a pregnant woman with eclampsia, coma, and/or convulsions without an alternate identifiable cause is one of the most common scenarios described in relation to PRES.

Eclampsia is seen in approximately 1% of births.^[7]^ Although most eclampsia occur between 20 weeks of pregnancy and 48 hours postpartum, some patients present eclampsia between 48 hours and 4 weeks postpartum.^[7–10]^ Thus, early postpartum eclampsia (taking place within 48 hours post-delivery) is distinct from late postpartum eclampsia, which occurs over 48 hours later.^[10,11]^ We experienced a patient with PRES and late onset postpartum eclampsia 10 weeks post-delivery. Late onset postpartum eclampsia concurrent with PRES is extremely rare; moreover, to the best of our knowledge, our case has the most delayed onset ever reported. Sharing our experience may increase awareness of PRES caused by delayed onset of eclampsia, which may help emergency physicians make early diagnoses and apply appropriate treatments.

### 1.1 Presentation of the case

Patient has provided informed consent for publication of the case. A previously healthy Asian 23-year-old woman (gravida one, para one) had a full-term pregnancy and delivered a healthy boy. During her puerperium and pregnancy period, she was normotensive without proteinuria, edema, or convulsion. She had no specific medical history or family history.

On postpartum day 71, she developed intermittent headache and nausea, which persisted for the following 2 days. Computed tomography (CT) revealed no abnormal findings.

On day 73, when she rose from bed, she complained of defocus on visual acuity, followed by disorder of consciousness and generalized tonic-clonic seizure associated with left conjugate deviation. After emergency medical service providers intubated and treated her with diazepam in a prehospital setting, the patient was moved to the emergency department. On admission, her vital signs were: blood pressure of 141/103 mm Hg, heart rate of 118 bpm, respiration rate of 12 breaths per minute, SpO_2_ of 98% (with manual ventilation), and body temperature of 36.4°C. Neurological findings were as follows: Glasgow Coma Scale score of E1VTM1, no anisocoria, and normal pupillary light reflex. No subsequent seizure or conjugate deviation was presented.

**Figure 1:**
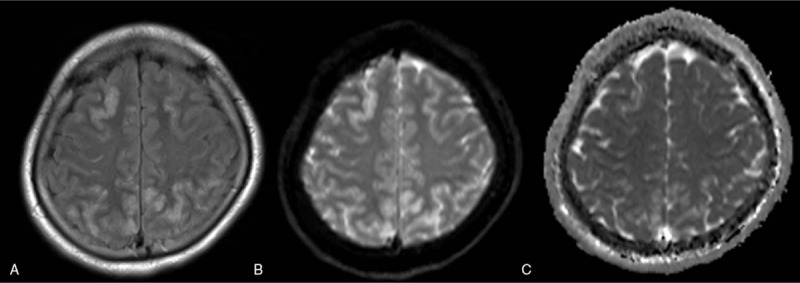
Radiographical findings on admission day. MRI axial T2-weighted FLAIR (A), DWI (B), and ADC mapping (C) demonstrated high-density areas in the parieto-occipital and frontal lobes. ADC = apparent diffusion coefficient, DWI = diffusion-weighted magnetic resonance imaging, FLAIR = fluid-attenuated inversion recovery, MRI = magnetic resonance imaging.

Brain MRI T2-weighted fluid-attenuated inversion recovery, apparent diffusion coefficient (ADC), and diffusion-weighted magnetic resonance (MR) imaging (DWI) mapping showed hyperintense cortical foci in the bilateral parieto-occipital lobe and frontal lobe. Cerebral vein and sinus MR angiography was normal, and no spasm is presented. (Fig. 1).

Lumbar puncture showed 1 lymphocyte/μL and cerebrospinal fluid protein of 86 mg/dL. Beta-2-microglobulin, angiotensin-converting enzyme (ACE), and *Mycobacterium tuberculosis* DNA were negative. Blood examination presented leukocytosis (11,220/μL), low folic acid (2.10 ng/mL), and hypopotassemia (2.9 mmol/L). No serological evidence of acute infection with the viruses listed in Table 1 were found. ACE and β-D glucan were negative either. Autoimmune antibodies listed in Table 2 were all negative. Treatment with levetiracetam (500 mg × 2/day) and edaravone (30 mg/day) was started on the day of admission. She became alert and oriented on the same day. After extubation, she complained of a slight headache and gaze nystagmus, but no visual disturbance, visual field defect, anisocoria, or parareflexia was presented. No motor or sensory paralysis was found. No hypertension or protein urea was noted either.

**Table 1:**
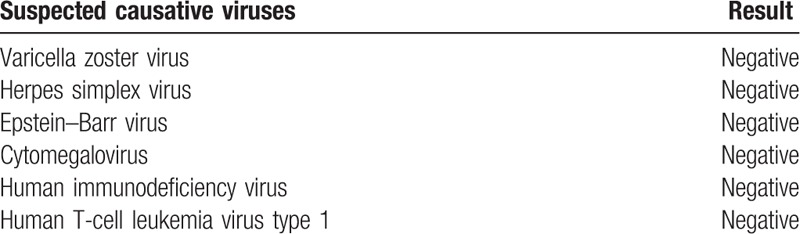
The viruses which were suspected as the cause of encephalitis. No serological evidence of acute infections were found.

**Table 2:**
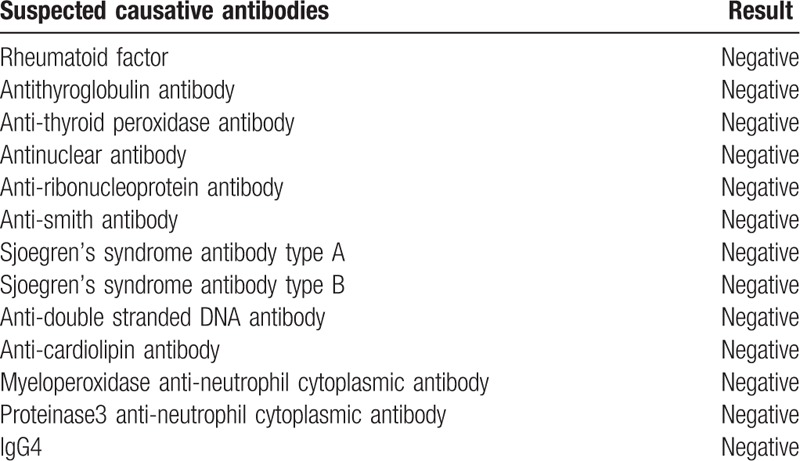
The antibodies which were suspected as the cause of autoimmune encephalitis. The results were all negative.

**Figure 2:**
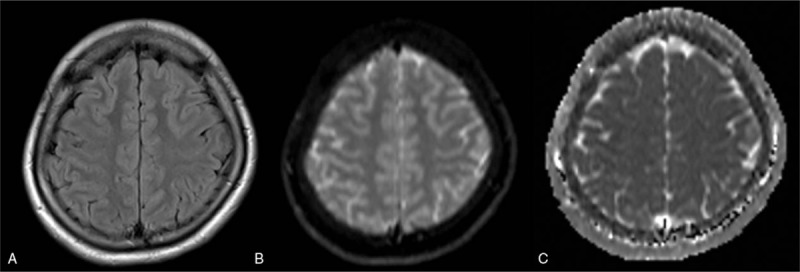
One month after admission. MRI axial T2-weighted FLAIR (A), DWI (B), and ADC mapping (C) showed complete remission of the foci. ADC = apparent diffusion coefficient, DWI = diffusion-weighted magnetic resonance imaging, FLAIR = fluid-attenuated inversion recovery, MRI = magnetic resonance imaging.

No further seizures occurred during the patient's hospital stay. Two days later, follow-up MRI showed a decrease of the foci. Electroencephalogram showed approximately 10 c/s alpha wave dominantly at the occipital lobe, and slow wave slightly burst at the frontal lobe. There was no significant epileptic discharge. The gaze nystagmus decreased that day. One month after, the foci had completely disappeared from both the DWI and ADC map. (Fig. 2)

## 2 Discussion and conclusion

PRES is associated with various factors such as hypertensive encephalopathy, renal failure, acute intermittent porphyria, thrombotic thrombocytopenic purpura, pre-eclampsia, and eclampsia.^[1]^ Our patient did not have any of these factors besides presumed eclampsia. However, we made the clinical diagnosis of PRES secondary to eclampsia based on her clinical course, the exclusion of other presumable diseases, and the hallmark radiological findings on CT and MRI.

The pathogenesis of PRES is still not fully understood, although it seems to be linked to failure of local blood flow autoregulation and endothelial dysfunction causing blood–brain barrier destruction and vasogenic edema.^[2,5,12,13]^ Close contact between autonomic neuron varicosities and smooth muscle cells is relatively sparse on the vertebrobasilar arteries (posterior circulation) compared to the anterior circulation. Therefore, it is presumed that the posterior circulation is more easily affected, with breakdown of the sympathetic autoregulation. Little has been revealed about the neurochemical phenotypes of the parasympathetic innervation of the vertebrobasilar arteries. Interestingly, nitric oxide (NO) synthase is predominantly distributed on the posterior cerebral arteries, particularly the basilar artery.^[14]^ The sympathetic activation of nitrergic vasodilation increases blood flow in the basilar artery in order to meet the need during the “fight-or-flight” response.^[15,16]^ Owing to this response, excess blood flow might be supplied to the occipitoparietal area, possibly resulting in the predilection region of PRES.

Eclampsia's pathophysiology is also not yet properly understood. Various factors such as modified immune mechanisms, placental abnormalities, oxidative stress, dysfunction of endothelial cells, dietary characteristics, and genetic susceptibility are thought to play a role in the pathogenesis of eclampsia.^[17]^ Eclampsia is also associated with an anatomical substrate recognized by neuroimaging as PRES. Radiologically, postpartum eclampsia consistently presents hyperintense foci in the occipital-parietal lobe on T2-weighted MRI. In eclampsia, seizures, headache, vision loss, and altered sensorium are the telltale signs of PRES.^[7,18,19]^ Focal neurological deficits besides cortical blindness are uncommon.

While PRES due to other causes is frequently accompanied by hypertension, high blood pressure (over 120 mm Hg diastolic) has been seen in only 20% of pre-eclampsia and eclampsia patients. Since auto-regulatory capacity is impaired during pregnancy, eclampsia can happen without hypertension.^[4,20,21]^ All basal arteries in patients with pre-eclampsia and eclampsia show increased blood flow velocity. Hyperperfusion and blood–brain barrier leakage can cause colloid drainage to the cerebral parenchyma, resulting in vasogenic edema and neuron damage.^[3,12]^

Patients with eclampsia and PRES may have concomitant metabolic abnormalities like liver enzyme irregularities, raised serum creatinine, hypomagnesemia, raised lactate dehydrogenase, deceased platelets, decreased serum albumin, raised cerebrospinal fluid albumin, and higher proteinuria,^[2,22]^ which were not observed in the present case.

Both PRES and eclampsia are caused by a vasoregulation disorder and an increase in blood flow, especially in the vertebrobasilar arteries. This leads to vasogenic edema, and severe cases could eventually progress to cytotoxic edema, some of them with irreversible neuropathy. Differentiating vasogenic edema from cytotoxic edema is possible with DWI and ADC mapping. Cytotoxic edema decreases ADC and shows a bright signal on DWI because it increases the intracellular fluid and decreases the extracellular fluid. In contrast, vasogenic edema increases ADC and shows no signal on DWI, or it can be visualized as an increased signal by the T2 shine-through effect. Increased ADC values correspond with highly mobile water in vasogenic edema. However, when the 2 types of edema are combined, restricted diffusion in cytotoxic edema decreases ADC values while vasogenic edema increases them. As a result, the responses cancel each other out.^[23]^

Equally important, PRES needs to be differentiated from other diseases including cerebral venous sinus thrombosis or ischemic stroke. In the present case, since the foci were high signals on DWI and ADC, it might have been combined edema or only vasogenic edema. Considering her post-treatment course, it was more likely to be the latter.

Clinical and radiographic characteristics are usually reversible. Seizures usually never happen when radiological abnormalities resolve. Medication is no longer necessary at this point, but delayed treatment might result in permanent brain damage.

Our case demonstrates that postpartum eclampsia can happen even 10 weeks after delivery. Appropriate differential diagnosis of the diseases associated with PRES enables physicians to make an early diagnosis and initiate proper treatment, which may result in complete reversibility of the condition. The present case is quite rare because our patient might have presented extremely late onset postpartum eclampsia.

## Author contributions

**Conceptualization:** Toshiyuki Aokage, Atsunori Nakao.

**Data curation:** Kaori Masai, Yayoi Ueda, Hiromichi Naito.

**Supervision:** Hiromichi Naito.

**Writing – original draft:** Kaori Masai, Atsunori Nakao.

**Writing – review & editing:** Yayoi Ueda, Hiromichi Naito, Kohei Tsukahara, Toshiyuki Aokage, Noritomo Fujisaki, Hirotsugu Yamamoto, Atsunori Nakao.
